# Chronic diseases and multi-morbidity in persons experiencing homelessness: a cross-sectional study

**DOI:** 10.1093/eurpub/ckac131.123

**Published:** 2022-10-25

**Authors:** W Lutchmun, G Froeschl, J Gach, C Bader, C Borup

**Affiliations:** 1 Center for International Health, Ludwig-Maximilians-Universitaet, Munich, Germany; 2 Division of Infectious Diseases, University Hospital LMU, Munich, Germany; 3 Aerzte der Welt Deutschland, e.V., Munich, Germany

## Abstract

**Background:**

Persons experiencing homelessness (PEH) suffer a high burden of chronic diseases, yet face significant barriers in accessing health services. We describe and compare chronic diseases and multi-morbidity in PEH, housing exclusion and secure housing who lacked access to regular health services in the wake of the COVID-19 pandemic in Germany.

**Methods:**

Study participants were adults who sought care at clinics of the humanitarian organisation “Ärzte der Welt” in Munich, Hamburg and Berlin in 2020. The patients were categorised into three groups according to the European Typology of Homelessness and Housing Exclusion (ETHOS). We described socio-demographic characteristics, self-rated health, chronic diseases, multi-morbidity and SARS-COV-2 infections in each group. Logistic regression analysis was used to identify socio-demographic factors associated with higher odds of chronic diseases and multi-morbidity.

**Results:**

Of the 695 study participants, 333 experienced homelessness, 292 housing exclusion and 70 had secure housing. 92.3 % had no or limited health insurance and 96.7% were below the poverty line. Males and EU citizens were highly represented among PEH (74.2% and 56.8% respectively). PEH had lower self-rated health (47.8%, p = 0.04), and higher rates of psychiatric illness (20.9%, p = 0.04). In adjusted analyses, being 35-49 and ≥50 years was associated with greater odds of chronic diseases (AOR= 2.33, 95% CI = 1.68-3.24; AOR=3.57, 95% CI = 2.55-5.01, respectively), while being male was associated with lower odds of multi-morbidity (AOR=0.602, 95% CI = 0.38-0.9). Of the 18 symptomatic patients tested for SARS-COV-2 infection, 15 were PEH, of whom one tested positive.

**Conclusions:**

Housing was not a risk factor for chronic disease and multi-morbidity in this study. However, PEH reported poorer self-rated and psychiatric health. Strategies to improve access to health services for persons experiencing homelessness and housing exclusion are much needed in Germany.

**Key messages:**

• Research to highlight the health inequity, both in access and outcomes, in persons experiencing all forms of homelessness is much needed.

• This study provides data on a population disconnected from the formal healthcare system. Making such data visible is a step towards addressing the structural causes of social exclusion.

